# Mito-TEMPO Alleviates Renal Fibrosis by Reducing Inflammation, Mitochondrial Dysfunction, and Endoplasmic Reticulum Stress

**DOI:** 10.1155/2018/5828120

**Published:** 2018-03-25

**Authors:** Yuqing Liu, Yundan Wang, Wei Ding, Yingdeng Wang

**Affiliations:** Division of Nephrology, Shanghai Ninth People's Hospital, School of Medicine, Shanghai Jiao Tong University, 639 Zhizaoju Road, Shanghai 200011, China

## Abstract

**Background:**

Renal fibrosis is a common pathological symptom of chronic kidney disease (CKD). Many studies support that mitochondrial dysfunction and endoplasmic reticulum (ER) stress are implicated in the pathogenesis of CKD. In our study, we investigated the benefits and underlying mechanisms of Mito-TEMPO on renal fibrosis in 5/6 nephrectomy mice.

**Methods:**

Mice were randomly divided into five groups as follows: control group, CKD group, CKD + Mito-TEMPO (1 mg·kg^−1^·day^−1^) group, CKD + Mito-TEMPO (3 mg·kg^−1^·day^−1^) group, and Mito-TEMPO group (3 mg·kg^−1^·day^−1^). Renal fibrosis was evaluated by PAS, Masson staining, immunohistochemistry, and real-time PCR. Oxidative stress markers such as SOD2 activity and MDA level in serum and isolated mitochondria from renal tissue were measured by assay kits. Mitochondrial superoxide production was evaluated by MitoSOX staining and Western blot. Mitochondrial dysfunction was assessed by electron microscopy and real-time PCR. ER stress-associated protein was measured by Western blot.

**Results:**

Impaired renal function and renal fibrosis were significantly improved by Mito-TEMPO treatment. Furthermore, inflammation cytokines, profibrotic factors, oxidative stress markers, mitochondrial dysfunction, and ER stress were all increased in the CKD group. However, these effects were significantly ameliorated in the Mito-TEMPO treatment group.

**Conclusions:**

Mito-TEMPO ameliorates renal fibrosis by alleviating mitochondrial dysfunction and endoplasmic reticulum stress possibly through the Sirt3-SOD2 pathway, which sheds new light on prevention of renal fibrosis in chronic kidney disease.

## 1. Introduction

Chronic kidney disease (CKD) characterized by progressive and irreversible loss of renal function is a highly life-threatening public health issue worldwide [1, 2]. Chronic renal failure, the end stage of chronic kidney disease, is manifested by glomerulosclerosis, tubulointerstitial fibrosis, and vascular sclerosis [3]. Nevertheless, the pathophysiological mechanisms implicated in renal fibrosis are multifactorial, and the exact mechanism is not yet clear. It is most likely a combination of oxidative stress, inflammation, epithelial to mesenchymal transition, apoptosis, and extremely high deposition of extracellular matrix [4, 5].

The mitochondrion is an essential organelle crucial in energy generation, calcium homeostasis, reactive oxygen species (ROS) production, and cell apoptosis [6]. Mitochondrial dysfunction is implicated in many diseases through impaired ATP production, accumulated mitochondrial DNA (mtDNA) damage, and increased ROS production [7]. Mitochondrial dysfunction plays a vital role in the pathogenesis and the development of various forms of CKD [8]. In the last few years, mitochondria-target therapeutics have attracted much interest as it has been demonstrated that attenuating mitochondrial dysfunction restrains the progression of kidney diseases [9, 10]. Therefore, mitochondria are a potent therapeutic target in preventing CKD progression.

The endoplasmic reticulum (ER) is essential for not only protein biosynthesis and posttranslational modification process but also for calcium storage, lipid biosynthesis, detoxification, energy metabolism, and reduction-oxidation (redox) balance [11]. The process of protein folding in the ER is extremely sensitive to intracellular and extracellular stimuli, and accumulation of unfolded and misfolded proteins in the ER lumen induces ER stress and activate the unfolded protein response (UPR) to help resolve ER stress. However, if the ER stress is too severe or persistent or if the UPR is impaired, the apoptotic signaling pathways are triggered [12]. Many studies have demonstrated that ER stress plays a crucial role in the progression of renal disease [13, 14]. Furthermore, recent work has indicated that mitochondrial dysfunction may be a contributing factor to ER stress [15, 16]. However, the specific mechanism remains to be elucidated.

Mito-TEMPO, a kind of mitochondria-targeted superoxide mimetic, contains piperidine nitroxide TEMPOL conjugated with a positively charged triphenylphosphonium cation which facilitates 1000-fold accumulation into the mitochondrial matrix [17]. Recent studies demonstrated that treatment with Mito-TEMPO protects mice against doxorubicin-induced cardiotoxicity by ameliorating mitochondrial dysfunction [18]. In addition, a previous study indicated that delayed therapy with Mito-TEMPO mitigates sepsis-induced mitochondrial dysfunction contributing to improved renal function and survival rate [19]. Furthermore, our previous studies have shown that Mito-TEMPO prevents aldosterone-induced renal tubular cell injury by improving mitochondrial dysfunction and inhibiting the activation of the NLRP3 inflammasome and cell apoptosis [20]. Although its benefits in improving mitochondrial dysfunction have been reported in various studies [18–20], its effect on renal fibrosis in the 5/6 nephrectomy model has not yet been discussed. In this study, we investigated the benefits and mechanisms of mitochondria-targeted antioxidant Mito-TEMPO on renal fibrosis in 5/6 nephrectomy mice.

## 2. Materials and Methods

### 2.1. Reagents and Antibodies

Mito-TEMPO and MitoSOX were purchased from Sigma-Aldrich (St. Louis, MO, USA). Antibodies against CHOP, BiP, GRP94, *β*-actin, and HRP-conjugated secondary antibodies were purchased from Cell Signaling Technology (Beverly, MA, USA). Antibodies against SOD2 and caspase-12 were purchased from Santa Cruz Biotechnology (Santa Cruz, CA, USA). Antibodies against Sirt3 and acetyl-K68-SOD2 were obtained from Abcam (Cambridge, MA, USA). T-SOD assay kit, SOD2 assay kit, MDA assay kit, ATP assay kit, and mitochondrial isolation kit were purchased from Beyotime Institute of Biotechnology (Jiangsu, China).

### 2.2. Animal Experiments

All animal experiments were performed with the approval of the Animal Care Committee at Jiao Tong University. C57BL/6J (wild-type (WT)) male mice were purchased from Shanghai SLAC Laboratory Animals (Shanghai, China). The animals were kept in a temperature-controlled animal facility on a 12-hour light/dark cycle, with water and food ad libitum. 5/6 nephrectomy was performed in the mice (weighing 22–25 g) through two stages of surgery as described previously [21]. Mice were randomly divided into five groups: (1) sham-operated mice were treated with physiological saline solution (control); (2) 5/6 nephrectomy mice were treated with physiological saline solution (CKD); (3) 5/6 nephrectomy mice were treated with Mito-TEMPO (1 mg·kg^−1^·day^−1^ ip) (CKD + MT1); (4) 5/6 nephrectomy mice were treated with Mito-TEMPO (3 mg·kg^−1^·day^−1^ ip) (CKD + MT3); and (5) sham-operated mice were treated with Mito-TEMPO (3 mg·kg^−1^·day^−1^ ip) (MT3). Mito-TEMPO was dissolved in physiological saline solution and kept in the dark at 4°C. Mice were treated with Mito-TEMPO at a dose of 1 mg·kg and 3 mg·kg by intraperitoneal (ip) injection every day. After 12 weeks, the mice were sacrificed, blood samples were collected, and sections of kidney tissue was fixed in 4% paraformaldehyde for histological evaluation. The remainder of the kidney was snap-frozen in liquid nitrogen for subsequent protein and mRNA analysis.

### 2.3. Serum Biochemical Measurements

Serum creatinine and blood urea nitrogen (BUN) were measured by standard laboratory methods. Furthermore, serum malondialdehyde (MDA) and serum total superoxide dismutase (T-SOD) were detected using commercial kits (Beyotime Institute of Biotechnology). The absorbance was read at 450 nm (T-SOD) and 533 nm (MDA), according to the manufacturer's instructions.

### 2.4. Histological Examination and Immunohistochemistry

Renal tissues were sectioned into 3 *μ*m slices and stained with periodic acid-Schiff reagent (PAS) and Masson's trichrome. Glomerular damage was quantified according to published methods [22]. The severity of tubulointerstitial damage was graded according to interstitial collagen deposition using Masson's trichrome staining [23].

Immunohistochemistry was performed to detect the expression and localization of the proteins of interest. Paraffin-embedded sections of the renal cortex were stained with anti-fibronectin (FN) (1 : 250) and anti-TGF-*β* (1 : 500) antibody prior to hematoxylin counterstaining. The expression of FN and TGF-*β*1 in the entire cortex (a cross-section of the kidney) was determined using an image analyzer version 6.1 (WinROOF), and the data were expressed as a percentage of the positive area examined.

### 2.5. Mitochondrial Isolation and Purification

Remove the kidney tissue capsules and chop finely, wash and rinse the blood water, dry with filter paper, and cut into small pieces. Add 1.0 mL ice precooling of Lysis Buffer and homogenize using the loose-fitting pestle on ice for 10 passes. Quickly transfer the homogenate into centrifuge tubes and centrifuge for 5 min at 10000*g* at 4°C. The supernatant was taken to new centrifuge tubes and centrifuge for 5 min at 10000*g* at 4°C. The supernatant was taken and centrifuge for 10 min at 12000*g* at 4°C. The resulting supernatant was removed and the pellet fraction containing mitochondria was further resuspended with wash buffer. After centrifugation at 12000*g* at 4°C for 10 min, the supernatant was collected as a mitochondrial fraction.

### 2.6. SOD-2 Activity and MDA Measurement

The SOD-2 activity and MDA level in renal mitochondrial fraction were determined using commercial kits (Beyotime Institute of Biotechnology). All experimental procedures were performed according to the manufacturer's instructions. Microplate assay was used to detect the absorbance of the sample at 450 nm (SOD2) and 533 nm (MDA).

### 2.7. Mitochondrial Superoxide Measurement

Mitochondrial ROS in kidney tissues was detected with MitoSOX™ Red reagent. Briefly, renal tissues were sectioned into 10 *μ*m slices and put in the wet box. 5 mM MitoSOX was diluted with PBS (1 : 500) and then 10 *μ*M Hoechst was diluted with previous diluted MitoSOX solution (1 : 500). Add a mixture of 150 *μ*l to the kidney on each slice and incubate for 40 min protecting from light at 37°C. Wash three times with PBS and mounted onto microscope slides. Images were taken with Nikon fluorescent microscope (excitation/emission maxima: ~510/580 nm). MitoSOX fluorescence intensity was quantified using Image Pro Plus software.

### 2.8. Electron Microscopy

To visualize the ultrastructural morphology of mitochondria, kidney tissue samples were cut into 1 mm^3^ pieces using a scalpel and fixed with 2.5% glutaraldehyde at room temperature. Ultra-thin sections (60 nm) were prepared using a microtome. The sections were then placed on copper grids and stained with uranyl acetate and lead citrate for evaluation by electron microscopy.

### 2.9. Detection of ATP

The ATP levels in the mouse kidney were determined using a commercial ATP assay kit (Beyotime Institute of Biotechnology) based on the luciferin-luciferase reaction. Kidney tissues (20 mg) were lysed and centrifuged at 12,000*g*, 4°C for 5 min. 20 *μ*l supernatants were then mixed with 100 *μ*l detection working solution in a black 96-well plate. Then, the chemiluminescence was measured.

### 2.10. Western Blot

Renal tissue was lysed in protein lysis buffer on ice, and protein concentration was measured. Equal amounts of proteins in each sample were loaded onto 10% SDS-PAGE gels. Proteins were then transferred to PVDF membranes, and the membranes blocked with 5% nonfat dry milk in Tris-buffered saline-Tween20 (TBST) for 2 h. Then, the membranes were incubated overnight with antibodies against Sirt3, SOD2, AcSOD2, CHOP, BiP, GRP94, and *β*-actin at a dilution of 1 : 1000. After washing with TBST, the blots were incubated with secondary antibodies for 1 h at room temperature. The immune complexes were visualized with an enhanced chemiluminescent system (Amersham, Little Chalfont, Bucks, UK), and densitometric analysis was performed using Quantity One software (Bio-Rad, Hercules, CA, USA).

### 2.11. Real-Time PCR

Total RNA was extracted from kidney tissue using TRIzol reagent (Invitrogen, Carlsbad, CA, USA). Reverse transcription using Transcriptor First Strand cDNA Synthesis Kit (Takara, Japan) according to the manufacturer's instruction. RT-PCR was performed using the SYBR Green Master Mix on the ABI Prism 7500 Sequence Detection System (Foster City, USA). Comparative 2^−ΔΔCt^ method was used to determine the relative quantification of the target gene, with GAPDH as an internal reference. The primer sequences used for PCR amplification are summarized in Table 1.

### 2.12. Statistical Analysis

All data are presented as the mean ± standard deviation (mean ± SD). Comparisons among groups were made by one-way analysis of variance (ANOVA) followed by Tukey's post hoc test. *P* < 0.05 was considered to be statistically significant.

## 3. Results

### 3.1. Mito-TEMPO Attenuates Body Weight Loss and Impaired Renal Function in 5/6 Nephrectomy Mice

**Figure 1:**
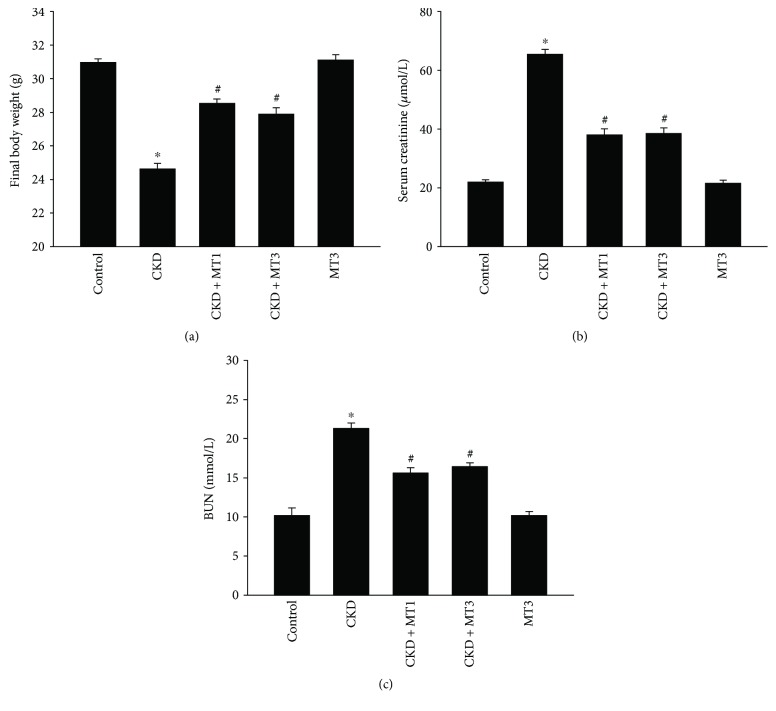
Evaluation of biochemical indexes in mice at the end of 12-week study. (a) Final body weight, (b) serum creatinine, and (c) BUN. Data are presented as mean ± SD (*n* = 6). ^∗^*P* < 0.01, control group versus CKD group; ^#^*P* < 0.01, CKD group versus CKD + MT1 and CKD + MT3 groups. BUN: blood urea nitrogen.

Figure 1(a) demonstrates that the final body weight of mice in the CKD group was significantly reduced compared to controls (24.83 ± 0.33 g versus 30.93 ± 0.23 g). Compared with the CKD group, the final body weight in CKD + MT1 and CKD + MT3 groups (28.23 ± 0.47 g and 28.17 ± 0.45 g, resp.) was significantly improved, although still lower than control mice. Similarly, as shown in Figures 1(b) and 1(c), the concentration of serum creatinine and BUN was significantly higher in the CKD group (65.5 ± 5.15 *μ*mol/L and 21.35 ± 2.27 mmol/L, resp.) compared to control group (22 ± 1.77 *μ*mol/L and 10.2 ± 2.27 mmol/L, resp.). Compared with the CKD group, serum creatinine and BUN concentrations were significantly decreased in the CKD + MT1 group (38.14 ± 5.15 *μ*mol/L and 15.64 ± 1.86 mmol/L, resp.) and CKD + MT3 group (38.57 ± 4.79 *μ*mol/L and 16.45 ± 1.33 mmol/L, resp.). Furthermore, there was no statistical significance between the CKD + MT1 group and the CKD + MT3 group for final body weight, serum creatinine, or BUN concentrations (Figures 1(a)–1(c)).

### 3.2. Mito-TEMPO Alleviates Renal Fibrosis in 5/6 Nephrectomy Mice

**Figure 2:**
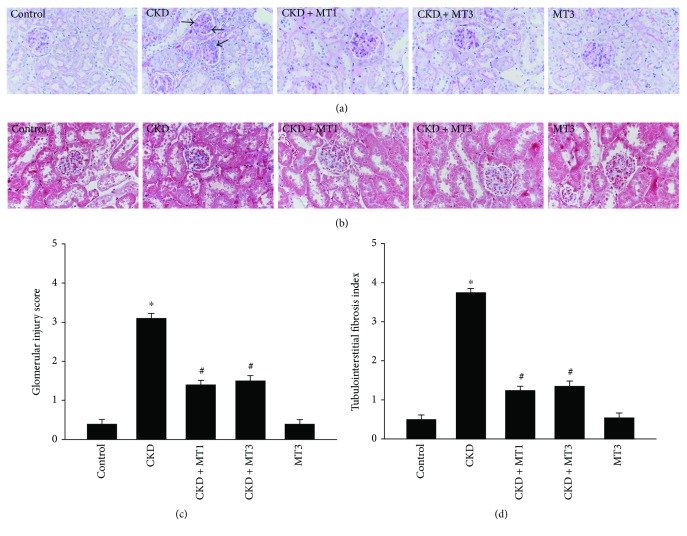
Kidney histopathology. Representative photomicrographs of PAS-stained kidney sections (a) (magnification, ×400) and Masson's trichrome-stained kidney sections (b) (magnification, ×400). Glomerular injury score (c) and tubulointerstitial fibrosis index (d) were assessed as previously described in Materials and Methods. Data are presented as mean ± SD (*n* = 6). ^∗^*P* < 0.01, control group versus CKD group; ^#^*P* < 0.01, CKD group versus CKD + MT1 and CKD + MT3 groups.

Renal histopathology was examined by PAS staining and Masson staining (Figures 2(a)–2(d)). The CKD mice manifested severe glomerular injury characterized by cell proliferation, accumulated extracellular matrix, and glomerulosclerosis (Figure 2(a)). Compared with the CKD group, the glomerular injury score was significantly reduced in the CKD + MT1 and CKD + MT3 groups (Figure 2(c)). Similarly, Figure 2(b) shows the representative micrographs of kidney sections following Masson staining. Compared with the CKD group, treatment with Mito-TEMPO (CKD + MT1 and CKD + MT3 groups) markedly improved renal interstitial collagen accumulation. Furthermore, quantitative determination by computer-aided morphometric analyses revealed that treatment with Mito-TEMPO reduced renal interstitial collagen accumulation and renal fibrotic lesions in 5/6 nephrectomy mice (Figure 2(d)).

**Figure 3:**
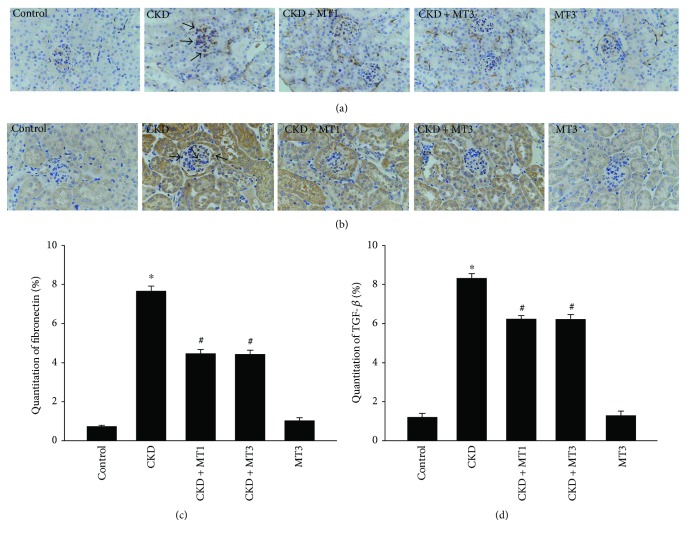
Immunohistochemical staining of fibronectin (a) (magnification, ×400) and TGF-*β* (b) (magnification, ×400) in kidney sections. Semiquantitative analysis of fibronectin (c) and TGF-*β* (d) positive areas are evaluated according to immunohistochemical staining. Data are presented as mean ± SD (*n* = 6). ^∗^*P* < 0.01, control group versus CKD group; ^#^*P* < 0.01, CKD group versus CKD + MT1 and CKD + MT3 groups.

Renal immunohistochemical analysis of fibronectin and TGF-*β* is presented in Figures 3(a)–3(d). The fibronectin and TGF-*β*-positive areas in the CKD group are significantly elevated compared to the control group, demonstrating renal fibrosis in the 5/6 nephrectomy model. Significant reversal of these effects was observed in both the CKD + MT1 and CKD + MT3 groups.

**Figure 4:**
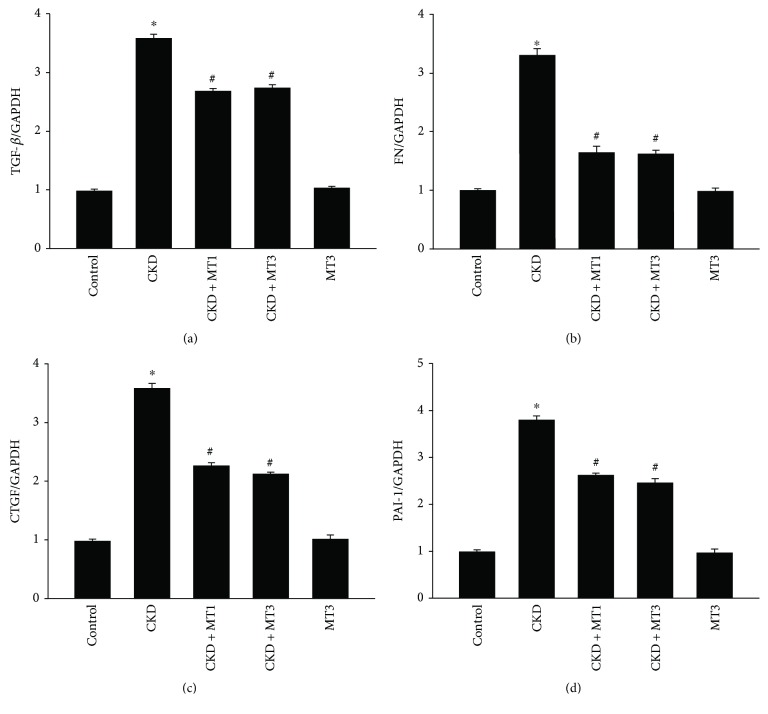
Expression of renal fibrosis indicators in the kidney. Semiquantitative analysis of renal TGF-*β* (a), FN (b), CTGF (c), and PAI-1 (d) mRNA expression normalized to GAPDH detected by real-time PCR. Data are presented as mean ± SD (*n* = 6). ^∗^*P* < 0.01, control group versus CKD group; ^#^*P* < 0.01, CKD group versus CKD + MT1 and CKD + MT3 groups.

Indicators of renal fibrosis measured by real-time PCR are shown in Figures 4(a)–4(d). Renal TGF-*β*, FN, CTGF, and PAI-1 mRNA levels were significantly increased in the CKD group (3.6-fold, 3.3-fold, 3.6-fold, and 3.8-fold, resp.) compared to the control group. TGF-*β*, FN, CTGF, and PAI-1 mRNA levels significantly declined in the CKD + MT1 and CKD + MT3 groups relative to the CKD group.

### 3.3. Mito-TEMPO Inhibits 5/6 Nephrectomy-Induced Expression of Inflammatory Cytokines

**Figure 5:**
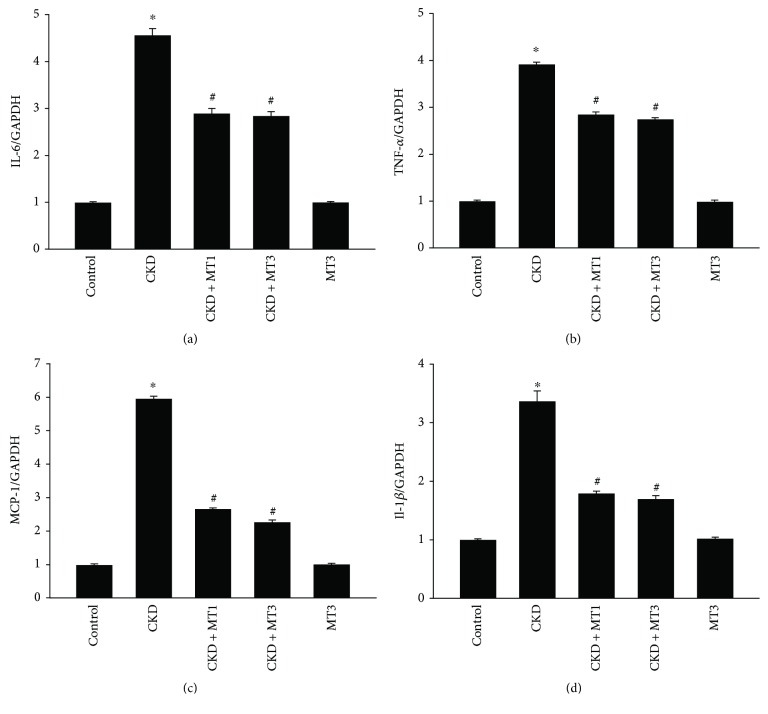
The effect of Mito-TEMPO on 5/6 nephrectomy-induced expression of inflammatory cytokines. Semiquantitative analysis of renal IL-6 (a), TNF-*α* (b), MCP-1 (c), and IL-1*β* (d) mRNA expression normalized to GAPDH detected by real-time PCR. Data are presented as mean ± SD (*n* = 6). ^∗^*P* < 0.01, control group versus CKD group; ^#^*P* < 0.01, CKD group versus CKD + MT1 and CKD + MT3 groups.

The expression of 5/6 nephrectomy-induced inflammatory cytokines was evaluated by real-time PCR in Figures 5(a)–5(d). IL-6, TNF-*α*, MCP-1, and IL-1*β* mRNA levels were significantly raised in the CKD group (4.6-fold, 3.9-fold, 6.0-fold, and 3.4-fold, resp.) compared to the control group. However, these effects were suppressed by Mito-TEMPO treatment. Indeed, mRNA levels of IL-6, TNF-*α*, MCP-1, and IL-1*β* significantly decreased in the CKD + MT1 and CKD + MT3 groups compared to the CKD group.

### 3.4. Mito-TEMPO Suppresses 5/6 Nephrectomy-Induced Oxidative Stress

**Figure 6:**
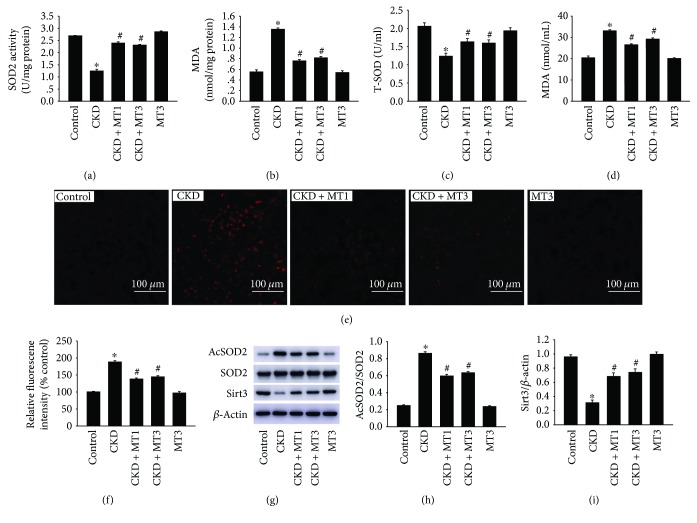
The effect of Mito-TEMPO on 5/6 nephrectomy-induced oxidative stress. The SOD2 activity (a) and MDA level (b) in isolated kidney mitochondria, serum T-SOD (c), and MDA (d) levels were measured following the manufacturer's protocol. Mitochondrial superoxide was detected by MitoSOX Red fluorogenic dye and images were taken with Nikon fluorescent microscope (e) (magnification, ×200), and the mean MitoSOX fluorescence intensity per image was calculated by Image Pro Plus software and normalized to the control group (f). Western blot analysis of Sirt3, SOD2, and AcSOD2 (g). Relative expression of AcSOD2 (h) normalized to the expression of SOD2 and Sirt3 (i) normalized to the expression of *β*-actin. Data are presented as mean ± SD (*n* = 6). ^∗^*P* < 0.01, control group versus CKD group; ^#^*P* < 0.01, CKD group versus CKD + MT1 and CKD + MT3 groups.

Oxidative stress was detected by determination of SOD2 activity, MDA level in mitochondrial fractions and serum MDA, and T-SOD levels. The SOD2 activity in isolated kidney mitochondria was significantly decreased in the CKD group (1.24 ± 0.07 U/mg protein) compared to the control group (2.69 ± 0.02 U/mg protein) (Figure 6(a)). Compared to the CKD group, administration of Mito-TEMPO remarkably ameliorated the SOD2 activity in the CKD + MT1 and CKD + MT3 groups (2.4 ± 0.05 U/mg protein and 2.31 ± 0.03 U/mg protein) (Figure 6(a)). The similar results were obtained in the serum T-SOD level (Figure 6(c)). Similarly, the MDA level in isolated kidney mitochondria was significantly increased in the CKD group (1.35 ± 0.26 nmol/mg protein) compared to the control group (0.55 ± 0.39 nmol/mg protein) (Figure 6(b)). Treatment with Mito-TEMPO prevented oxidative stress in the CKD + MT1 and CKD + MT3 groups (0.76 ± 0.26 nmol/mg protein and 0.82 ± 0.26 nmol/mg protein, resp.) relative to the CKD group (Figure 6(b)). In addition, there are similar results in the serum MDA level (Figure 6(d)). No statistical significance was measured between the CKD + MT1 and CKD + MT3 groups. Moreover, we confirmed the presence of mitochondrial superoxide to evaluate the source of CKD-induced ROS. The fluorescence intensity of MitoSOX increased remarkably in the CKD group but markedly reduced by treatment with Mito-TEMPO (Figures 6(e) and 6(f)).

To confirm that increased ROS production can contribute to decreased Sirt3 expression, impaired SOD2 activity, and increased SOD2 acetylation, we analyzed the expression of Sirt3 and SOD2 acetylation in the kidney by Western blot (Figures 6(g)–6(i)). The SOD2 acetylation (3.46-fold) is significantly increased in the CKD group compared to the control group (Figures 6(g) and 6(h)). In addition, the expression of Sirt3 is sharply decreased in the CKD group compared to the control group (Figures 6(g) and 6(i)). However, these effects were restrained by Mito-TEMPO treatment.

### 3.5. Mito-TEMPO Attenuates 5/6 Nephrectomy-Induced Mitochondrial Dysfunction

**Figure 7:**
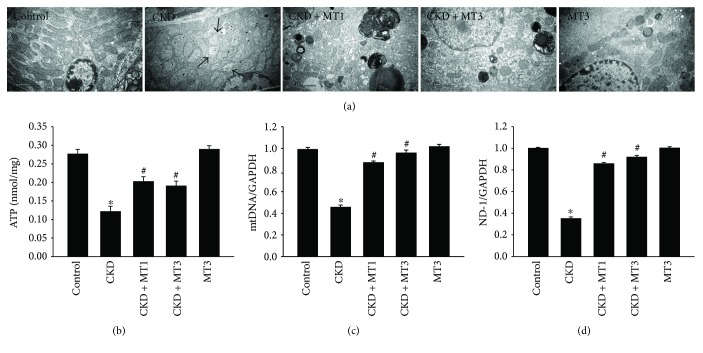
Mito-TEMPO ameliorates 5/6 nephrectomy-induced mitochondrial dysfunction. Representative electron microscopy photomicrographs of ultrastructural morphology of mitochondria (a) (magnification, ×10,000). Arrow indicates swollen mitochondria. The ATP levels (b) in the kidney were determined according to the manufacturer's protocol. Semiquantitative analysis of renal mtDNA (c) and ND-1 (d) mRNA expression normalized to GAPDH detected by real-time PCR. Data are presented as mean ± SD (*n* = 6). ^∗^*P* < 0.01, control group versus CKD group; ^#^*P* < 0.01, CKD group versus CKD + MT1 and CKD + MT3 groups.

Mitochondrial dysfunction is characterized by impaired intracellular ATP synthesis, increased mtDNA damage, and excessive ROS production. The ultrastructural morphology of renal cells reveals swollen mitochondria with disorganized and fragmented cristae in the CKD group (Figure 7(a)). This phenotype is accompanied by significantly reduced ATP level, mtDNA, and ND-1 mRNA levels in the renal tissue of CKD group compared with the control group (Figures 7(b)–7(d)). However, Mito-TEMPO treatment alleviates mitochondrial dysfunction in the CKD + MT1 and CKD + MT3 groups compared with the CKD group (Figures 7(a)–7(d)). In addition, there was no statistical significance between CKD + MT1 and CKD + MT3 groups.

### 3.6. Mito-TEMPO Ameliorates 5/6 Nephrectomy-Induced ER Stress

**Figure 8:**
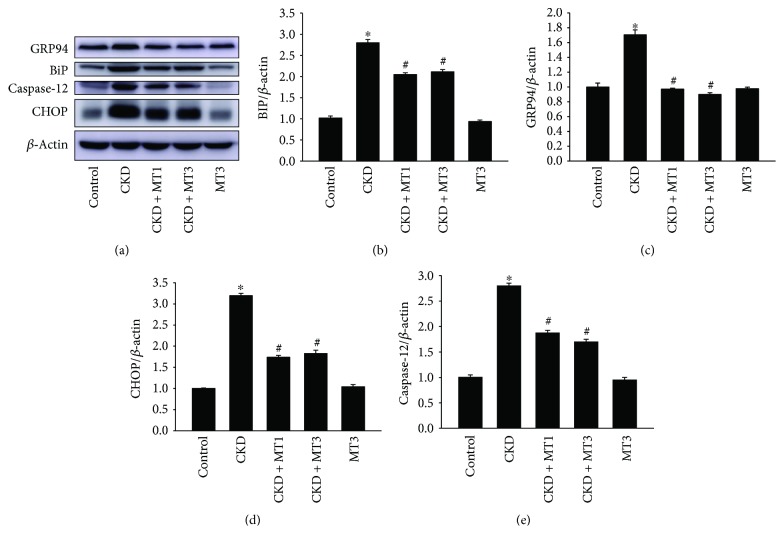
Mito-TEMPO attenuates 5/6 nephrectomy-induced ER stress. Western blot analysis of CHOP, caspase-12, BiP, and GRP94 (a). Relative expression of BiP (b), GRP94 (c), CHOP (d), and caspase-12 (e) normalized to the expression of *β*-actin. Data are presented as mean ± SD (*n* = 6). ^∗^*P* < 0.01, control group versus CKD group; ^#^*P* < 0.01, CKD group versus CKD + MT1 and CKD + MT3 groups.

Increased ROS production increases the level of unfolded proteins in the ER and contributes to ER stress. BiP and GRP94 are ER stress markers regulated by ER function. The expression of BiP (2.2-fold) and GRP94 (1.7-fold) measured by Western blot are increased in the CKD group compared to the control group (Figures 8(a)–8(c)). However, these effects were restrained by Mito-TEMPO treatment. The levels of BiP and GRP94 in renal tissue were markedly decreased in the CKD + MT1 and CKD + MT3 groups compared to the CKD group (Figures 8(a)–8(c)). When ER stress is too severe, apoptosis can be activated via CHOP and caspase-12 signal pathways. The expression of CHOP (3.2-fold) and caspase-12 (2.8-fold) was significantly increased in the CKD group relative to the control group (Figures 8(a), 8(d), and 8(e)). Mito-TEMPO treatment remarkably reduced the levels of CHOP and caspase-12 (Figures 8(a), 8(d), and 8(e)). This indicates that CHOP and caspase-12 signaling is crucial in 5/6 nephrectomy-induced renal injury. Furthermore, there was no significant difference between CKD + MT1 and CKD + MT3 groups.

## 4. Discussion

Renal fibrosis is a major and severe consequence of CKD progression, contributing to poor prognosis. Many studies indicate that inflammation, mitochondrial dysfunction, and ER stress are implicated in the progression of renal diseases [8, 14]. However, the specific pathophysiological mechanisms of renal fibrosis of CKD are unknown. Mito-TEMPO, a mitochondria-targeted superoxide mimetic, has been demonstrated to prevent the loss of renal function by attenuating mitochondrial dysfunction [19, 20]. In the present study, our results suggest that Mito-TEMPO attenuates the 5/6 nephrectomy-induced renal fibrosis by ameliorating inflammation, mitochondrial dysfunction, and ER stress. Therefore, mitochondrial dysfunction-mediated ER stress accounts for the renal fibrosis induced by 5/6 nephrectomy model.

In this report, the 5/6 nephrectomy model served to investigate the mechanisms and possible therapies of renal fibrosis. We determined that the physiological parameters, including final body weight and renal function (serum creatinine and BUN), were significantly decreased in 5/6 nephrectomy mice but improved by Mito-TEMPO administration. Based on renal histopathology and immunohistochemical analysis, renal fibrosis, including glomerulosclerosis and tubulointerstitial damage, was apparent in 5/6 nephrectomy mice. However, these effects were reversed by Mito-TEMPO treatment at both the histopathological and transcriptional level. Furthermore, our results showed that the inflammatory cytokines IL-6, TNF-*α*, MCP-1, and IL-1*β* were increased significantly in the 5/6 nephrectomy group but significantly decreased following Mito-TEMPO administration. Therefore, we conclude that inflammation can contribute to the 5/6 nephrectomy-induced renal fibrosis.

Alterations in cell organelle structure, such as mitochondria and endoplasmic reticulum, generally correlate to changes in function. Mitochondrial dysfunction is characterized by decreased ATP production, accumulated mitochondrial DNA damage, and increased ROS production. Excessive oxidative stress is responsible for the mitochondrial dysfunction leading to activation of apoptotic pathways. Mitochondrial DNA, a second cellular genome, encodes the essential mitochondrial proteins of the respiratory chain [24]. ND-1, a subunit of mitochondrial complex I, is encoded by mtDNA. The lack of ND-1 causes mitochondrial dysfunction by disrupting the stability of complex I, complex IV, and the respiratory supercomplex [25]. ATP, the energy source for basic cell functions and cellular repair and regeneration, is generated in the mitochondria via oxidative phosphorylation. Decreased ATP production is an important marker for mitochondrial dysfunction. Many studies have demonstrated that mitochondrial dysfunction is central in the progression of CKD [8, 26]. In this study, we report severe mitochondrial dysfunction in the 5/6 nephrectomy group, which exhibit swollen mitochondria, decreased ATP production, and reduced mtDNA and ND-1 copy number. This further supports that mitochondrial dysfunction is a contributing factor in the induction of renal fibrosis in the 5/6 nephrectomy model. Furthermore, our results also demonstrate that Mito-TEMPO attenuates 5/6 nephrectomy-induced mitochondrial dysfunction.

In our study, oxidative stress markers AcSOD2 and MDA and mitochondrial superoxide were increased significantly in the 5/6 nephrectomy group but significantly decreased following Mito-TEMPO administration. These effects were attenuated by Mito-TEMPO treatment, most likely due to its superoxide and alkyl radical scavenging properties [17, 27]. As far as we know, there are three kinds of superoxide dismutase (SODs) localized in mammalian cells. Among them, mitochondrial SOD2 is thought to be the major antioxidant enzyme-scavenging cellular ROS [28]. Previous reports demonstrated that SOD2 activity was strongly regulated by acetylation at several conserved lysine residues, and Sirt3 plays an important role in keeping SOD2 deacetylation and activity, which leads to ROS reduction and protection against excessive oxidative stress [29, 30]. Moreover, it has also been pointed out that preventing Sirt3 and SOD2 activity from being reduced may help to block increased oxidative stress in a remnant kidney [31]. Therefore, application of SOD2 mimetic may be beneficial for kidney injury. In this study, we found out that Sirt3 expression and SOD2 activity were reduced in renal tissue, followed by increased mitochondrial ROS, which were all notably reversed by administration of Mito-TEMPO. This is consistent with the previous report in terms of the role of Mito-TEMPO [30]. We may conclude that Mito-TEMPO reduced mitochondrial ROS and relieve mitochondrial dysfunction through Sirt3-SOD2 pathway in an injured kidney.

The endoplasmic reticulum is a cellular organelle mainly involved in protein biosynthesis, posttranslational modification, calcium storage, and reduction-oxidation balance [11]. Numerous reports have demonstrated that ER stress is involved in the onset of some kidney diseases, including glomerular epithelial cell injury induced by renal ischemia-reperfusion [32] and podocyte injury induced by the accumulation of advanced oxidation protein products [33], and tubular epithelial cell death in aldosterone-induced renal injury [34]. Furthermore, many studies have revealed the interactions between UPR and hypoxia, oxidative stress, or inflammation. BiP and GRP94, ER molecular chaperones, serve as ER stress markers and central regulators of the UPR and participate in modulating the folding process [35]. CHOP is a member of the CCAAT/enhancer-binding protein (C/EBP) family of transcription factors implicated in ER stress-induced apoptosis [36]. In the present study, we demonstrated that expression of BiP and GRP94 was significantly increased in the CKD group. However, this was reversed by Mito-TEMPO treatment. The expression of BiP and GRP94 was markedly reduced in the CKD + MT1 and CKD + MT3 groups. Failure of adaptive UPR due to severe or prolonged ER stress leads to apoptosis via CHOP and caspase-12-mediated apoptotic pathways. Accordingly, in our study, the expression of CHOP and caspase-12 were significantly higher in the CKD group. However, Mito-TEMPO administration remarkably suppressed the expression of CHOP and caspase-12. These results indicate that ER stress was involved in 5/6 nephrectomy-induced renal injury by activating CHOP and caspase-12 signaling. Furthermore, the reversal effects of Mito-TEMPO treatment suggest that mitochondrial dysfunction is a contributing factor in ER stress, indicating the organelle crosstalk between mitochondria and endoplasmic reticulum.

The mitochondrion and endoplasmic reticulum are two vital organelles that contribute to cell apoptosis. Recent studies suggest that mitochondria and endoplasmic reticulum do not function independently but rather interact with each other in several ways. However, the molecular mechanisms controlling this interaction are still unclear [37, 38]. Previous studies have suggested apoptotic signals usually originate from the ER and signal to the mitochondria. However, few studies have reported apoptotic signals traveling in the reverse direction, from the mitochondria to the ER. Reactive oxygen species contribute to ER stress, a process called ROS-mediated ER stress. When mitochondrial dysfunction occurs, increased ROS production results in unfolded protein response in the ER, thus inducing ER stress, which if sustained, can lead to apoptosis [39, 40]. Iwasawa et al. suggested that when mitochondrial dysfunction occurs, the mitochondrial fission protein, fission 1 homologue (Fis1), serves to transmit an apoptotic signal from the mitochondria to the ER by interacting with Bap31 at the ER [41]. Similarly, our results suggest that mitochondrial dysfunction-mediated ER stress is involved in the 5/6 nephrectomy-induced renal fibrosis.

Mito-TEMPO, which functions as a mitochondrial-specific superoxide mimetic, has been tested in vitro in our previous study [20]. In addition, in a proximal tubular epithelial cells ATP depletion-recovery (ATP-DR) injury model, Mito-TEMPO treatment partially preserves mitochondrial membrane integrity and attenuates ATP-DR mediated necrosis and apoptosis by preventing mitochondrial permeability transition and decreasing Bax translocation to the mitochondria [42]. However, its effect on 5/6 nephrectomy-induced renal fibrosis has not yet been reported. We are the first to report that Mito-TEMPO alleviates renal fibrosis in 5/6 nephrectomy mice by attenuating inflammation, mitochondrial dysfunction, and ER stress.

## 5. Conclusions

In conclusion, our study demonstrates that mitochondrial dysfunction, inflammation, and ER stress are involved in 5/6 nephrectomy-induced renal fibrosis. Furthermore, Mito-TEMPO attenuates renal injury by ameliorating inflammation, mitochondrial dysfunction, and ER stress. Mito-TEMPO can serve as a promising therapeutic in suppressing the progression of renal fibrosis in CKD patients.

## Figures and Tables

**Table 1:** RT-PCR primer sequences.

Gene	Forward primer sequence (5′–3′)	Reverse primer sequence (5′–3′)
TGF-*β*	TACCATGCCAACTTCTGTCTGGGA	TGTGTTGGTTGTAGAGGGCAAGGA
CTGF	GGGCCTCTTCTGCGATTTC	ATCCAGGCAAGTGCATTGGTA
PAI-1	TTCAGCCCTTGCTTGCCTC	ACACTTTTACTCCGAAGTCGGT
FN	GCAGTGACCACCATTCCTG	GGTAGCCAGTGAGCTGAACAC
mtDNA	TTTTATCTGCATCTGAGTTTAATCCTGT	CCACTTCATCTTACCATTTATTATCGC
ND-1	ATCCTCCCAGGATTTGGAAT	ACCGGTAGGAATTGCGATAA
MCP-1	GTTGGCTCAGCCAGATGCA	AGCCTACTCATTGGGATCATCTTG
IL-6	TAGTCCTTCCTACCCCAATTTCC	TTGGTCCTTAGCCACTCCTTC
TNF-*α*	CCCTCACACTCAGATCATCTTCT	GCTACGACGTGGGCTACAG
IL-1*β*	TGCACTACAGGCTCCGAGAT	CGTTGCTTGGTTCTCCTTGT
GAPDH	AGGTCGGTGTGAACGGATTTG	TGTAGACCATGTAGTTGAGGTCA
